# Case Report: Gastric-Type Endocervical Adenocarcinoma Mimicking Submucosal Myoma Under Hysteroscopy

**DOI:** 10.3389/fmed.2022.845445

**Published:** 2022-03-08

**Authors:** Jiao Wang, Qing Yang, Dandan Wang, Mengyuan Li, Ningning Zhang

**Affiliations:** Department of Obstetrics and Gynecology, Shengjing Hospital of China Medical University, Shenyang, China

**Keywords:** endocervical adenocarcinoma, gastric-type, human papillomavirus, submucosal myoma, diagnosis

## Abstract

Gastric-type endocervical adenocarcinoma (GAS) is considered a distinct and clinically important entity because it is unrelated to human papillomavirus infection and has aggressive behavior and worse clinical outcomes than the usual type of endocervical adenocarcinoma (ECA). The preoperative diagnosis of GAS is often difficult because of its nonspecific clinical manifestations and special lesion location. We report the case of a 50-year-old Chinese woman who presented with intermittent left lower abdominal pain for 1 year. Preoperative images showed left hydrosalpinx and a lesion that was mainly located in the lower part of the uterine cavity. We considered the lesion to be a polyp before surgery. During hysteroscopic surgery, we suspected that it may be a submucosal myoma. However, pathology revealed that it was a GAS. GAS may be located in the upper endocervix or even reach the uterine cavity. The appearance is occasionally similar to that of submucosal myoma, resulting in difficult preoperative diagnosis and even misdiagnosis.

## Introduction

Cervical cancer is the fourth most common gynecological malignancy worldwide (1). Most cervical cancers are related to the continuous infection of high-risk human papillomavirus (HPV). However, a few are not, such as some endocervical adenocarcinoma (ECA). ECA accounts for 20–25% of invasive cervical cancers, and its incidence is gradually increasing (2). In the 2020 World Health Organization (WHO) classification of female genital tumors, ECAs are subclassified into human papillomavirus (HPV)-associated (HPVA) and HPV-independent (HPVI) groups based on their distinct etiology and clinical behavior (3). The most common histological type of the HPVI group is gastric-type ECA (GAS) (4), which has a worse prognosis than HPVA (5). The clinical manifestations are not specific, the focus is hidden, and the positive rate of screening and biopsy is low, which brings great challenges to timely and correct diagnosis. Here, we present a patient with GAS with lower uterine cavity involvement, which was misdiagnosed as submucosal myoma during hysteroscopic surgery. However, pathology confirmed the diagnosis of GAS.

## Case Report

**Figure 1:**
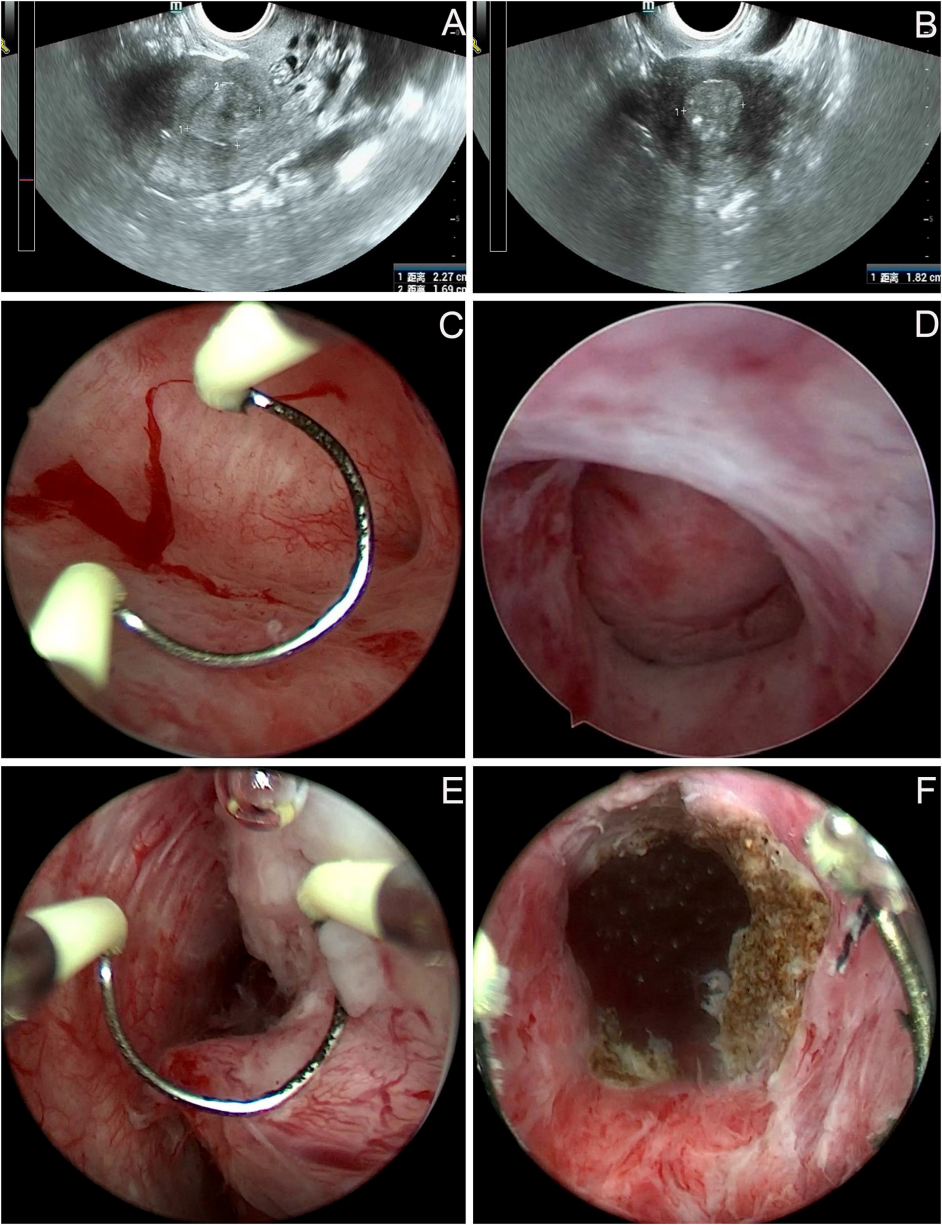
Imaging findings of the patient before and during the first operation. **(A,B)** Pelvic ultrasound: a mass around 2.3 × 1.8 × 1.7 cm was seen in the lower part of uterine cavity. The internal echo was uneven, and the boundary was clear. **(C–F)** Hysteroscopy: a mass with a size of about 2.5 × 2.0 cm was seen at the left anterior wall close to the internal os. The local shape was irregular, the surface contained thickened blood vessels. The tumor was removed using a circular electrode, the boundary between the attachment position of tumor and cervical tissue was unclear.

The patient was a 50-year-old premenopausal female, gravida 2, para 1. Cesarean section followed by right adnexectomy, which was performed due to a right ovarian cyst, was performed in 1995. On June 15, 2021, the patient visited our hospital for “intermittent left lower abdominal pain for 1 year.” In May 2020, pelvic ultrasound was performed in another hospital because of left lower abdominal pain and fever. The results showed a cystic mass in the left adnexa, about 7.6 × 1.4 cm, with a high possibility of hydrosalpinx; an intrauterine device (IUD) and liquid dark area in the uterine cavity, and Nabothian cysts in the cervix. Her symptoms were relieved after anti-inflammatory treatment, but slight intermittent pain persisted in the lower abdomen. The patient underwent pelvic ultrasound again on May 26, 2021. The results showed left hydrosalpinx, about 7.5 × 3.4 cm, and in addition to the IUD and cervical Nabothian cysts, a slightly strong echo mass was seen in the uterine cavity. Then the patient was referred to our hospital. On gynecological examination, the vulva and vagina were normal, a small amount of secretion could be seen, the cervix was of normal size with a smooth surface, the uterus was normal, the left adnexa were thickened and accompanied by slight tenderness. Pelvic ultrasound revealed that a mass around 2.3 × 1.8 × 1.7 cm was seen in the lower part of uterine cavity. The internal echo was uneven, and the boundary was clear (Figures 1A,B). Color Doppler flow imaging detected blood flow signals. The size of the left ovary was normal, and a 7.7 × 3.6 × 1.6 cm cystic mass was seen in the left adnexal area, which was tortuous and tubular, with liquid inside and a clear boundary. Thinprep cytology test (TCT) revealed atypical squamous cells of undetermined significance (ASCUS); HPV: negative. The serum carbohydrate antigen (CA)-199, carcinoembryonic antigen (CEA), CA-125, and CA-724 levels were normal. Preoperative diagnoses included left hydrosalpinx and endometrial polyp. Laparoscopic left salpingectomy, removal of the IUD, and resection of the mass under hysteroscopy were performed on June 22, 2021. Laparoscopic exploration revealed a thickened left fallopian tube, with a size of about 7.0 × 2.0 cm, which surrounded the left ovary. The right adnexa were absent. The uterus was normal in size and regular in shape. After removing the left fallopian tube, the left ovary was exposed and appeared normal, and hysteroscopic surgery was subsequently performed. We first removed the T-shaped IUD. A mass with a size of about 2.5 × 2.0 cm was seen at the left anterior wall close to the internal os (Figures 1C,D). The texture was tough, the local shape was irregular, the surface contained thickened blood vessels (Figure 1E), and the mucosa of the cervical canal was smooth. The lesion was removed using a circular electrode (Figure 1F). All resected tissues were sent for pathology examination. The patient recovered and was discharged on the fourth postoperative day. Paraffin pathology results were as follows: well-differentiated GAS and left hydrosalpinx. We contacted the patient and asked her to return to our hospital for further treatment.

**Figure 2:**
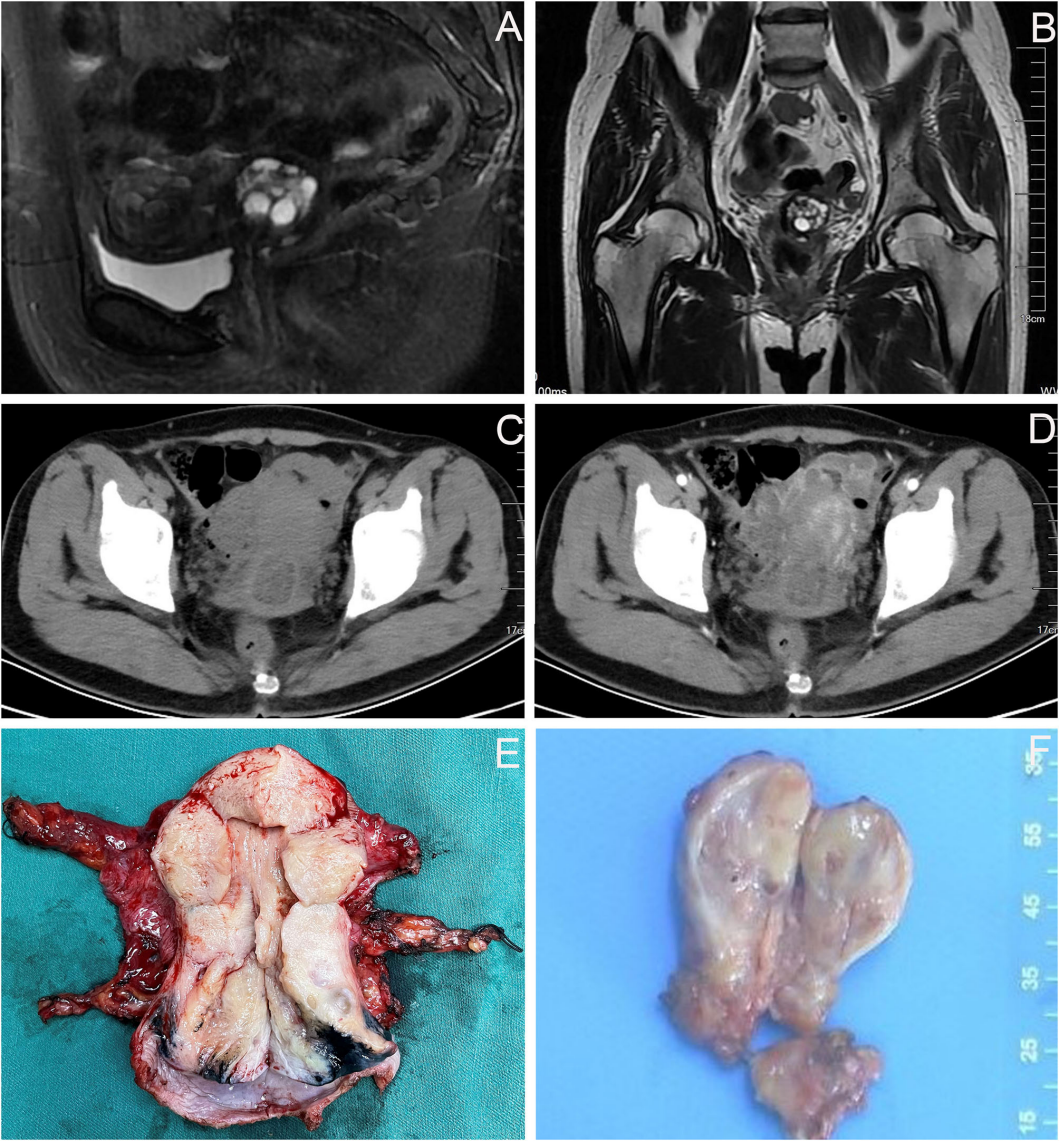
Imaging findings of the patient before and during the second operation. **(A,B)** Pelvic MRI: the cervix was plump, with an uneven equal T1 and slightly higher T2 signal shadow, which was mainly located in the posterior lip of the cervix, about 3.5 × 2.7 cm, in which there were many small circular long T1 and long T2 signal shadows. **(C,D)** Abdominal CT: the cervical was slightly plump, and multiple low-density small nodules could be seen in it (overall range of about 2.8 × 2.5 cm), and the enhancement was not obvious. **(E)** The cervix was slightly plump. **(F)** The appearance of the left ovary was normal, a tumor of approximately 1.5 × 1.3 cm was seen inside, the cut surface was yellowish white, multiple cystic cavities were seen around.

**Figure 3:**
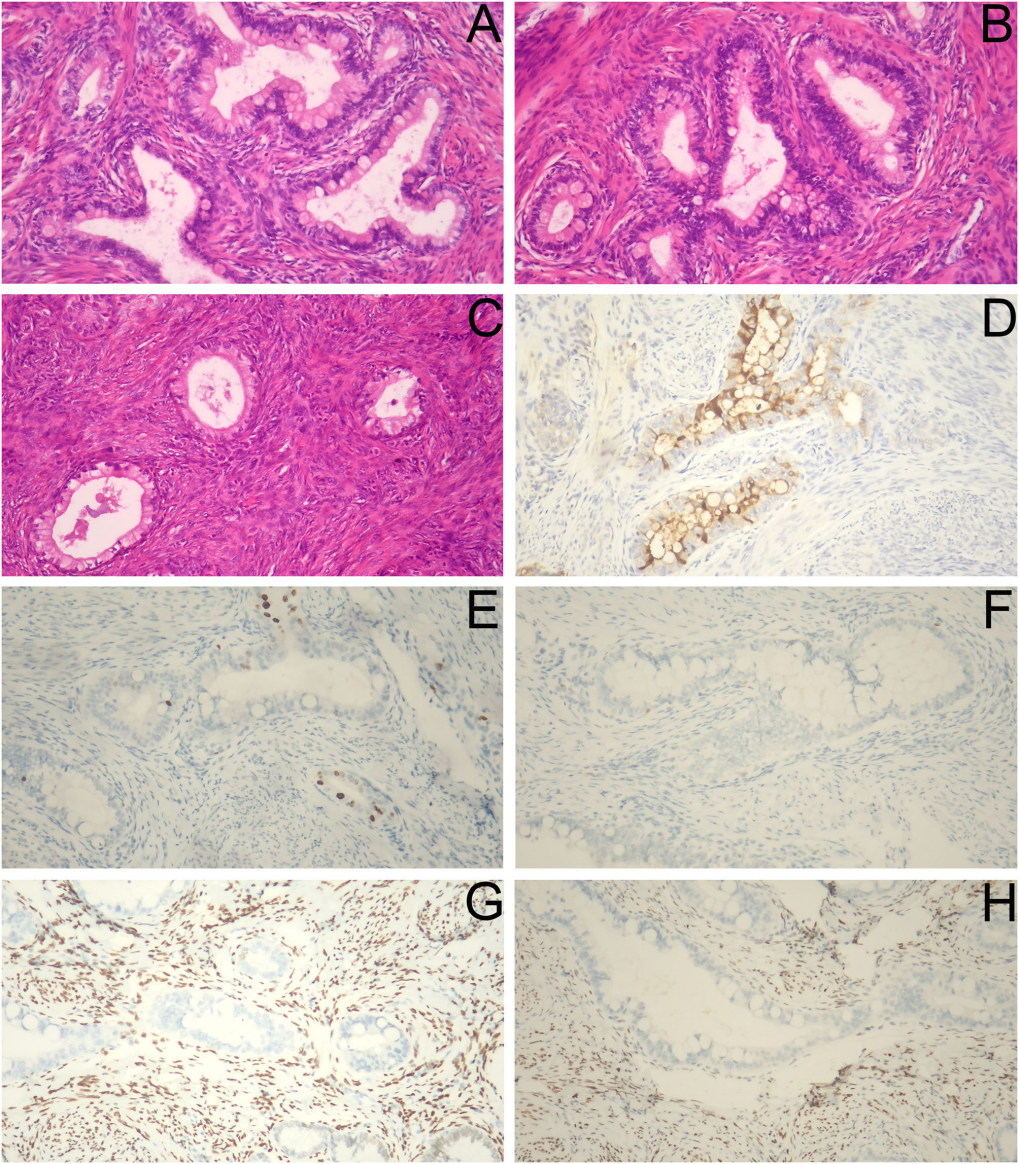
Histopathological and immunohistochemical staining findings. **(A,B)** The cancer cells were columnar and arranged in irregular glandular tubes, some nuclei were large and deeply stained, cytoplasm was empty and bright, and intracellular mucus were seen (H&E staining, ×40). **(C)** Heterotypic glands were seen in the left ovary (H&E staining, ×40). **(D)** CEA (focally +). **(E)** Ki-67 (about 5% +). **(F)** P16 (–). **(G)** ER (–). **(H)** PR (–) (**D–H**, ×100).

After the second admission, the patient's medical history was recorded. The patient said that she began having intermittent watery vaginal fluid approximately 1 year prior. She did not care for it or inform the doctors because she thought it was vaginitis. Gynecological examination showed that the cardinal and uterosacral ligaments were not thickened. Pelvic contrast-enhanced magnetic resonance imaging (MRI) showed that the cervix was plump, with an uneven equal T1 and slightly higher T2 signal shadow, which was mainly located in the posterior lip of the cervix. Weak enhancement was seen on contrast-enhanced scan, with a range of about 3.5 × 2.7 cm, in which there were many small circular long T1 and long T2 signal shadows (Figures 2A,B). No definite abnormality was found in the left ovary. Abdominal contrast-enhanced computed tomography (CT) showed that the cervix was slightly plump, and multiple low-density small nodules could be seen in it (overall range of about 2.8 × 2.5 cm), and the enhancement was not obvious (Figures 2C,D). Therefore, the diagnosis of stage IB2 GAS was made. Open operation was performed on July 26, 2021. During the operation, we found that the cervix was slightly plump (Figure 2E), the parauterine tissue was not thickened, and the appearance of the left ovary, peritoneal surface, greater omentum, and appendix were normal. First, the left ovary was removed and a tumor of approximately 1.5 × 1.3 cm was seen inside. The cut surface was yellowish white. Multiple cystic cavities were seen around (Figure 2F). The left ovary was subjected to frozen pathology, which suggested heterotypic glands and metastases. Therefore, radical hysterectomy, pelvic and paraaortic lymph node resection, greater omentum resection, and appendectomy were performed. There were no untoward events intraoperatively and postoperatively, and the patient was discharged on the eighth postoperative day. The paraffin pathological results were reported as follows: well-differentiated GAS, infiltrating the outer 1/3 of the cervical stroma; metastatic carcinoma of the left ovary; and no cancer at the surgical margin, parauterine, greater omentum, appendix, and lymph nodes. Microscopic examination revealed GAS (Figures 3A,B) and metastatic carcinoma of the left ovary (Figure 3C). Immunohistochemical analysis showed that the tumor was focally positive for CEA (Figure 3D), about 5% positive for Ki-67 (Figure 3E), and negative for P16, ER, and PR (Figures 3F–H). Four weeks after the second operation, the patient was referred to the gynecological tumor center for concurrent chemoradiotherapy (CCRT): pelvic intensity-modulated radiotherapy 50.4 Gy/28f + cisplatin concurrent chemotherapy. The patient completed the treatment, and there was no recurrence or metastasis during outpatient follow-up.

## Discussion

GAS, first described by Kojima et al. (6), was included as a subtype of HPVI group in the latest 2020 WHO classification of female genital tumors (3). It is an aggressive type of ECA characterized by mucinous morphology, gastric-type mucin, lack of association with HPV, and resistance to chemo/radiotherapy (7).

The incidence of GAS has obvious regional differences; GAS accounts for 10% of all ECA in an international population (4) and 20–25% of cases in Japanese women (8). Precursor lesions are essential for the prevention and early detection of cancer. Some findings suggest that atypical lobular endocervical glandular hyperplasia (LEGH) and gastric-type adenocarcinoma in *situ* are the precursors of GAS (9). D'Alessandro et al. (10) reported a case of GAS in presence of Nabothian cysts. They thought it would be interesting to evaluate the role of Nabothian cysts in the pathogenesis of GAS. The genetic underpinnings of GAS are beginning to be elucidated, as illustrated by the following recent studies. In the study by Park et al. (11) next-generation sequencing was performed in 21 GAS cases, and a total of 54 nonsynonymous somatic mutations were detected, with an average mutation rate of 2.6 per lesion. The mutated genes, of which TP53 was the most frequent, were mostly involved in signal transduction, DNA damage repair, and epithelial-mesenchymal transition. Another study showed that GAS most frequently harbored somatic mutations in TP53, CDKN2A, KRAS, and STK11, and potentially targetable mutations were identified in ERBB3, ERBB2, and BRAF (7).

The average age of patients with GAS is 49–51 years (6, 12). The symptoms of patients with GAS are diverse and non-specific. They are often situated in the upper endocervix, present with bleeding or profuse watery discharge, and are associated with a clinically bulky cervix without a well-defined mass due to an infiltrative growth pattern (13). Patients with GAS often have no specific signs of cervical cancer, and the surfaces of the cervix are mostly smooth. Most patients with GAS present at an advanced stage, and pelvic, abdominal, and distant metastases are not uncommon (5). Patients with ovarian and pelvic-abdominal metastases may have signs similar to those of ovarian cancer.

Screening methods for the usual cervical cancer are ineffective for GAS, which may result in a probable delay in diagnosis. Our case was initially treated in another hospital because of lower abdominal pain and fever. Ultrasound showed a hydrosalpinx, dark area in the uterine cavity, and cervical Nabothian cysts. Considering the abdominal pain and fever caused by hydrosalpinx, the patient was given anti-inflammatory treatment, while ignoring the performance of the dark area in the uterine cavity. Until this year, the patient did not undergo pelvic ultrasound again. In addition to hydrosalpinx, an abnormal mass in the uterine cavity was observed. Although TCT showed ASCUS, all high-risk HPVs were negative, and the possibility of an endometrial polyp was considered. During hysteroscopic surgery, we found that the tumor was located in the lower part of the uterine cavity near the internal os and considered that the possibility of submucosal myoma. However, the local shape of the tumor was irregular and like a fingertip; secondly, there was local vascular proliferation of the tumor; thirdly, multiple glandular cavities were seen on the section of the tumor; fourthly, after tumor resection, the capsule of myoma was not seen, and the boundary between the attachment position of tumor and cervical tissue was unclear. Hence, the diagnosis of GAS was accidentally determined based on the pathology. The diagnosis of GAS is primarily based on pathological morphology, combined with immunohistochemical examination if necessary (8). GAS is characterized by tumor cells showing clear, foamy, or pale eosinophilic cytoplasm and well-defined cytoplasmic borders, spanning the spectrum from well-differentiated to poorly differentiated tumors (7). Hysteroscopy is of great significance in the diagnosis of endometrial lesions. Ianieri et al. (14) developed a hysteroscopic scoring system according to some morphological and hysteroscopic parameters, which helps physicians, especially those less experienced, to make a differential diagnosis among normal endometrium, endometrial hyperplasia, and endometrial carcinoma. We believe that hysteroscopy also plays an important role in the diagnosis of cervical canal lesions because we can clearly observe the atypical vessels on the lesion in addition to the location of lesions, which can be useful in suspecting malignant lesions, as described in the report by Ianieri et al. and shown in our hysteroscopic images.

Patients with GAS may have elevated tumor markers, especially serum CA19-9 levels (15). However, the results of serum tumor markers in our case were all normal. Pelvic MRI features of “cosmos pattern” (CP), which means some small cysts or solid components are present in the central part of the lesion surrounded by relatively large cysts, can help to distinguish gastric-type mucin-positive lesions (GMPL) such as LEGH and GAS from gastric-type mucin-negative lesions (GMNL) such as cervical Nabothian cyst. If CP is observed as a hypointense area compared with the cervical stroma on T1WI, GMPL should be strongly suspected (16). A combination of cytology and MRI and an assay for gastric-type mucin have been suggested to be effective for the early detection and preoperative diagnosis of GAS (17). The differential diagnosis of GAS requires the inclusion of both benign and malignant lesions because of its complex and diverse morphologic features (12).

Clinical management can be challenging due to its rare incidence rate, cognitive limitations, diagnostic dilemmas, and aggressive behavior. Currently, there are no specific treatment guideline recommendations for GAS. Surgical removal of the uterus, adnexa, pelvic and/or paraaortic lymph nodes, omentum, appendix, and gross tumor might be considered because of its tendency to spread along surfaces throughout the peritoneal cavity and the higher likelihood of presenting with advanced stage (5). Our case confirmed the diagnosis of stage IB2 GAS after the second admission. Because GAS is prone to ovarian metastasis, we first removed the left ovary and sent it to intraoperative frozen pathology. The results suggested that heterotypic glands were observed, and metastasis was considered. During the first laparoscopic surgery, we did not find any abnormalities in the appearance of the left ovary. Radiologists also did not report any left ovarian abnormalities. This suggested that the ovarian metastatic lesions were insidious, and ovarian preservation was not recommended. Combined with the preoperative staging and intraoperative pathology, we performed radical hysterectomy and removed the paraaortic lymph nodes, greater omentum, and appendix. Postoperative paraffin pathology also revealed metastatic cancer of the left ovary. Although there were no high-risk factors for positive lymph nodes, positive margins, or positive parauterine status after surgical resection, CCRT was still recommended according to pathologic moderate risk factors of adenocarcinoma and invasion of the outer 1/3 cervical stroma.

GAS prognosis is significantly worse than that of UEA: the overall 5-year disease-specific survival rate of GAS is reportedly 30–42% compared to 74–91% for UEA (18). Progression-free survival and overall survival are poorer in patients with GAS than in those with UEA (8). The prognosis is related to tumor stage, parametrial invasion, surgical margin, metastasis, and treatment. Our patient completed the treatment and is in good condition without recurrence or metastasis.

There were some deficiencies in the diagnosis and treatment of our case. Although the patient's TCT suggested ASCUS, it did not attract enough attention because all high-risk HPVs were negative. Although some differences were found between the tumor and submucosal myoma during hysteroscopic surgery, the possibility of GAS was not considered, which led to another operation and increased the physical and economic burden. Due to economic reasons, the patient did not agree to undergo genetic testing.

## Conclusions

GAS is a type of HPV-independent ECA, which may be located in the upper endocervix or even reach the uterine cavity, resulting in a difficult preoperative diagnosis. Sometimes, the appearance is similar to submucosal myoma under hysteroscopy, which is easily misdiagnosed. MRI can show some characteristic changes and hysteroscopy can be used as a method to obtain pathological tissues for patients with GAS. Timely diagnosis is of great significance for the formulation of treatment plan and improving the prognosis of patients.

## Data Availability Statement

The original contributions presented in the study are included in the article/supplementary material, further inquiries can be directed to the corresponding author/s.

## Ethics Statement

The studies involving human participants were reviewed and approved by the Institutional Review Board of Shengjing Hospital of China Medical University (approval number: 2021PS849K). The patients/participants provided their written informed consent to participate in this study. Written informed consent was obtained from the individual(s) for the publication of any potentially identifiable images or data included in this article.

## Author Contributions

JW was responsible for the data collection and drafting of the manuscript. QY and DW participated in providing knowledge of the disease. ML analyzed the literature. NZ was responsible for critical revision of the manuscript. All authors read and approved the final manuscript.

## Funding

This work was supported by the Natural Science Foundation of Liaoning Province, China (no. 2021-MS-186), the 345 Talent Project of Shengjing Hospital, and the National Natural Science Foundation of China (no. 81872125).

## Conflict of Interest

The authors declare that the research was conducted in the absence of any commercial or financial relationships that could be construed as a potential conflict of interest.

## Publisher's Note

All claims expressed in this article are solely those of the authors and do not necessarily represent those of their affiliated organizations, or those of the publisher, the editors and the reviewers. Any product that may be evaluated in this article, or claim that may be made by its manufacturer, is not guaranteed or endorsed by the publisher.
